# Systematic screening as a tool for individualized rehabilitation following primary breast cancer treatment: study protocol for the ReScreen randomized controlled trial

**DOI:** 10.1186/s12885-020-06815-3

**Published:** 2020-05-29

**Authors:** U. Olsson Möller, L. Rydén, M. Malmström

**Affiliations:** 1 Department of Nursing and Integrated Health Sciences, Faculty of Health Sciences, Kristianstad University, Kristianstad, Sweden; 2 Department of Clinical Sciences Lund, Surgery, Lund University, Skåne University Hospital, Lund, Sweden; 3 Department of Health Sciences, Lund University, Box 157, 221 00, Lund, Sweden; 4 Department of Surgery and Gastroenterology, Skåne University Hospital, Lund, Sweden

**Keywords:** Breast cancer, Rehabilitation, Screening, RCT, Symptom distress, Sickness absence, Individualization

## Abstract

**Background:**

It is well known that women suffer from negative consequences following breast cancer (BC) treatment and that their largely varying needs for rehabilitation are often unmet. Up to 43% of these women are at risk of developing chronic distress requiring complex interventions; however, how to early identify and meet these women’s needs is unknown, leaving them with suboptimal chances of rehabilitation. The aim of the ReScreen study is to develop a model for and evaluate the effect of screening-based, individualized rehabilitation following primary BC treatment.

**Methods:**

The ReScreen study is designed as a complex intervention. Women with newly diagnosed BC are consecutively included in a three-armed randomized controlled trial. At inclusion, patients score their distress level on the Distress Thermometer (scale of 0–10) aiming to identify patients with extended rehabilitation needs. Patients scoring ≥5 are randomized to the intervention or control group while patients scoring ≤4 are followed longitudinally as an observational group. Patients in the intervention group, in conjunction with a dedicated research nurse, create an individualized rehabilitation plan based on an evidence-based decision support tool that was developed to create a solid base for the intervention. The research nurse will act as a continuous health care contact and be responsible for proactively and systematically evaluating patients’ needs to ensure that potential new problems or changed rehabilitation needs are identified throughout the 1-year follow-up period. The intervention will be evaluated through self-reported data focusing on physical and psychological outcomes as well as evaluation of satisfaction with care at baseline, 2 weeks and 3, 6, 9 and 12 months. Evaluation will also include health economic aspects based on register data and patients’ and relatives’ experiences of the rehabilitation process. In addition, optimal cut-off levels for distress as an indicator for extended rehabilitation needs will be investigated.

**Discussion:**

This study will provide important knowledge related to effectiveness of screening-based identification of rehabilitation needs and standardized evidence-based, individualized rehabilitation after primary BC treatment. With a complex intervention design, this study has the potential to form a comprehensive knowledge base which includes tools and guidelines for implementation into clinical practice.

**Trial registration:**

ClinicalTrials.gov NCT03434717. Registered February 15, 2018.

## Background

Despite extensive evidence of the positive effects of cancer rehabilitation, patients with breast cancer (BC) still suffer from unmet rehabilitation needs [1]. These patients are often affected physically as well as mentally but the level of constraints such as fatigue [2] or shoulder pain [3] and emotional distress [4] differs greatly between individuals, indicating a need for individualized rehabilitation.

Breast cancer is the most common cancer in women worldwide and accounts for about 28% of all cancer diagnosis [5], with approximately 7500 newly diagnosed cases in Sweden annually [6]. Decreased mortality rates have however, been reported [7, 8] and the 5- and 10- year survival rate in Sweden is as high as 92 and 86% respectively [6]. This means that a large number of women survive their cancer and may be in need of support and rehabilitation.

Breast cancer is associated with a wide range of persistent disabling complications such as pain [9], fatigue [2], depression [10], distress [11], fear of recurrence [12], psychosocial concerns [13] and reduced quality of life (QoL) [14]. It has been reported that, close to time of diagnosis, cancer patients in general suffer from unmet needs in terms of having someone to talk to, lack of information and education, and counselling relating to psychological, financial and occupational concerns [15]. Likewise, in women with BC, supportive care needs are associated with psychological concerns and information needs [16] and unmet needs are strongly associated with decreased QoL [17]. Studies have shown that 34–43% of newly diagnosed patients with BC suffer from high distress [11, 18] and are therefore at risk of developing chronic distress [18]. It has also been shown that 60% of women with BC report at least one adverse treatment effect as long as 6 years after the diagnosis [19]. Sequelae after BC have been shown to result in approximately 30–60% of women remaining on sickness absence 1 year after treatment [20, 21]. Patients’ ability to return to work and their work performance are associated with a combination of, and interaction between, occupational and work-related demands and environmental and individual factors [21], emphasizing the need for individualized rehabilitation also from a health economic perspective. Despite extensive knowledge about BC patients’ heterogeneous problems and needs, these women are often offered inadequate and non-individualized support and rehabilitation, leaving them with suboptimal chances of rehabilitation [1].

Rehabilitation is, together with diagnostics and treatment, the cornerstone of today’s BC trajectory and is fundamental to ensure optimized treatment effects and recovery. The medical treatment recommendations are based on evidence aiming to ensure optimal and equally high-quality care for all patients. However, evidence on cancer rehabilitation has to a large extent not been implemented in today’s cancer care. It has been reported that rehabilitation is often planned based on the novelty and severity of the diagnoses, with focus on treatment, side effects and care, which often draws the attention and focus away from survivorship care [22]. This despite the fact that medical indicators such as type of tumour have been shown to be modest indicators of distress and that poor QoL, disability, and unmet needs are more powerful predictors [23].

Early identification of women with extended support needs is a complex but fundamental task when aiming to ensure optimized rehabilitation and efficient use of available rehabilitation resources. Nevertheless, there is no consensus regarding how to identify women with specific or enhanced rehabilitation needs. Some studies have indicated that younger women experience greater psychological stress compared with older women [16, 24] and that not having a partner [1], advanced disease stage and shorter time since diagnosis are associated with a greater number of unmet needs [16]. But how to identify patients’ needs is often unclear and previous studies have shown that health care professionals (HCP) avoid structured assessments because of uncertainty and insufficient implementation, [25] or because they question the added value of screening tools [25, 26].

Distress has been shown to be a promising indicator to identify women with extended support needs during BC treatment [18, 24]. Distress is defined as a multi-factorial, unpleasant emotional experience of a psychological (cognitive, behavioural, emotional), social and/or spiritual nature that may interfere with the ability to cope effectively with cancer and its physical symptoms and treatments [27]. Earlier studies have shown that a cut-off of ≥7 on the 0–10-point Distress Thermometer [28] is relevant for identifying patients at risk of developing chronic distress [18]. However, these results need to be confirmed by larger-scale studies in various different contexts.

Research regarding support and rehabilitation for these patients often focuses on evaluating the effect of specific rehabilitation interventions on one or a few outcomes. Such research has provided solid evidence in respect of which rehabilitation interventions may be relevant, e.g. reporting positive effects of exercise on fatigue and physical functioning [29] or of yoga on anxiety and depression [30]. However, it has also been shown that one intervention may have positive effects on various different problems depending on the diverse array of aetiological causes underlying the problem, and on patients’ diverse preferences [31]. Based on this complexity it is prominent that studies focusing on specific interventions or problems have fallen short in terms of enabling the health care system to meet the varying and complex needs experienced by the patients, in an efficient and cost-effective way. Knowledge is therefore needed on how patients with extended needs can be identified and how the most effective rehabilitation interventions for each individual can be offered in a structured and systematic way.

## Methods

### Aim

The overall aim of the ReScreen study is to develop and evaluate a model for screening-based individualized rehabilitation for women following primary BC treatment. The project specifically aims to:

1. Evaluate the effect of screening-based individualized rehabilitation with focus on: (a) patients’ physical and psychological recovery; (b) patients’ satisfaction with the cancer trajectory/process; (c) health economic effects; and (d) patients’ and relatives’ experiences of the rehabilitation process.
2. Evaluate distress as an indicator for extended rehabilitation needs with focus on: (a) verifying the optimal cut-off score to identify patients with extended rehabilitation needs in Sweden; and (b) investigating the association between distress, demographic and medical data, and self-reported living habits.

### Design

**Fig. 1:**
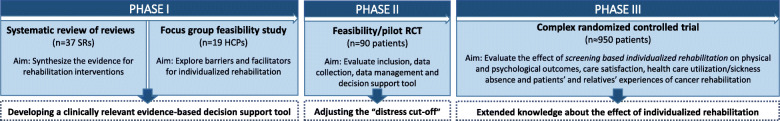
Description phase I-III. BC = breast cancer, HCP = Health care professionals, RCT = randomized controlled trial, SR = Systematic review

The ReScreen study is designed as a complex intervention study inspired by the Medical Research Council’s [32] guidelines for complex interventions. The overall project includes four phases, of which phase III, the randomized controlled trial (RCT), is the focus of this protocol. Phases I and II have been conducted aiming to determine the evidence base for the intervention as well as its feasibility, and to pilot test the RCT (described briefly below to provide an overall understanding of the project). The results of phase I–II have been used as a basis in the design of the full-scale RCT in phase III. The fourth phase, with focus on implementation and reporting, will be developed based on the results of phase I–III and is therefore not described in this protocol. An overview of phases I–III is presented in Fig. 1.

The complex intervention design is considered a process within which the work evolves through dialogue in order to develop, test and evaluate the intervention. This process is recommended when several factors are considered important in terms of change. A characteristic for complex interventions is that different methods/perspectives are often used to establish a comprehensive understanding of the problem, which aims to ensure clinical relevance and facilitate implementation [32].

To ensure clinical relevance a reference group has been formed, including both former BC patients and their relatives, and HCPs working with this group of patients. The group have been involved in the design of the intervention and the development of a decision support tool that is used as a basis for the intervention, and will be invited to participate throughout the project.

### Study setting

This RCT is conducted at a university hospital in southern Sweden at the Departments of Surgery and Oncology where approximately 670 patients annually are diagnosed with BC. At the Departments, patients are predominantly followed up based on the initial treatment regimen. Current rehabilitation practice includes access to a contact nurse who specifically works with BC patients. These nurses are the patients’ primary care contact and are available during the pre- and post- treatment phase. Additional rehabilitation resources are available depending on the treatment regimen and the patients’ needs and include, e.g., physiotherapists, occupational therapists and social workers. Rehabilitation in terms of follow-up is structured to some extent. For example, all patients with axillary lymph node dissection see a physiotherapist before and after surgery, focusing on lymphoedema prevention, while rehabilitation for patients without medically/treatment-induced follow-ups is based on the patients’ initiatives. Patients who are identified as having complex needs can be referred to a specialized cancer rehabilitation unit that includes a multi-professional team who exclusively focus on rehabilitation of patients with cancer.

Summary of Phase I-II: Identifying the evidence base and pilot and feasibility testing.

Phase I of this project has focused on developing an evidence-based, clinically relevant decision support tool, for individualized rehabilitation. This decision support tool aims to guide the research nurse responsible for the intervention through clarifying the evidence base for individualized rehabilitation. The content of the decision support tool is described in detail under “Intervention group” (below). As a first step in phase I, a systematic review of systematic reviews (SRs) [31] was conducted to explore the scientific foundation for rehabilitation following BC diagnosis. The review included 37 SRs and showed solid positive effects of exercise, physical activity and yoga, and yielded extended knowledge of the effects of complementary alternative medicine, lymphoedema treatment and psychosocial interventions.

As a second step, focus group interviews were conducted to illuminate HCPs’ experiences of barriers to and facilitators for individualized rehabilitation [33]. Nineteen HCPs from various professions representing surgery, oncology and specialized cancer rehabilitation were included and data were analysed using conventional qualitative content analysis [34]. The study identified individual and organizational barriers and facilitators in the cancer process that guide the intervention in phase III (RCT) (unpublished data).

Phase II included pilot and feasibility testing of the RCT, focusing on data collection and data management, as well as evaluating the decision support tool developed in phase I. A total of 90 patients were included in the pilot study. Based on preliminary analyses of the randomization procedure (i.e. based on the baseline distribution of the Distress Thermometer score), the initial cut-off of ≥7, which was based on previous research [11, 18], was changed to ≥5 for the full-scale study to ensure sensitivity and enable an evaluation of the optimal cut-off in the Swedish BC context (unpublished data).

### Phase III: evaluating the RCT

#### Design

**Fig. 2:**
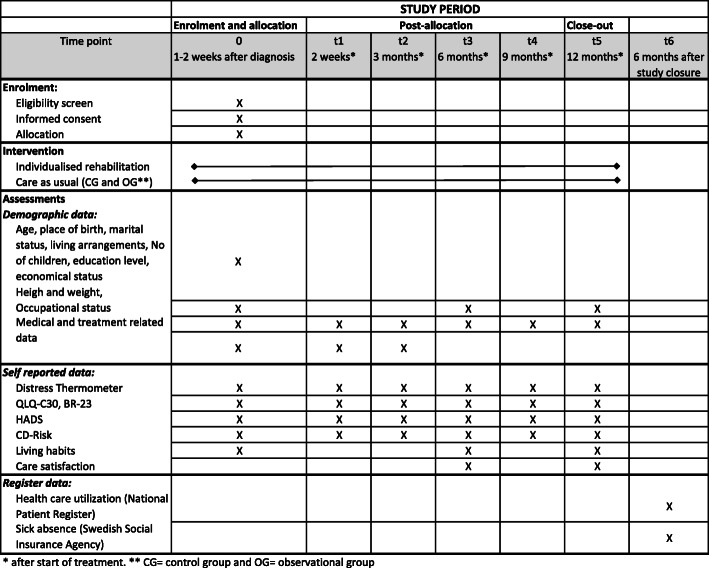
SPIRIT flow diagram of the ReScreen study

The study is designed as a complex prospective RCT evaluating the effects of screening-based, individualized rehabilitation following primary BC treatment. The study was initially started as a single-centre study but will, during the research process, be extended to a multi-centre study to enhance recruitment rate and generalizability. The reporting of the protocol will follow the SPIRIT guidelines (Additional file) and a brief structured summary of the trial is shown in a WHO trial registration data set (Table 1) and a SPIRIT flow diagram (Fig. 2).

**Table 1:** WHO trial registration data set for the ReScreen study

Data category	Information
Primary registry and trial identifying number	ClinicalTrials.gov: NCT03434717
Date of registration in primary registry	February 15, 2018
Secondary identifying numbers	None
Source(s) of monetary or material support	Skåne University Hospital, Department of Surgery and Gastroenterology
Primary sponsor	Skåne University Hospital, Department of Surgery and Gastroenterology
Secondary sponsor(s)	N/A
Contact for public queries	MM. RN. PHD. Skåne University Hospital, Department of Surgery and Gastroenterology 22,185 Lund, Sweden
Contact for scientific queries	MM. RN. PHD. Skåne University Hospital, Department of Surgery and Gastroenterology 22,185 Lund, Sweden
Public title	Optimized rehabilitation following primary breast cancer surgery - Systematic screening as a tool for individualized rehabilitation
Scientific title	Optimized rehabilitation following primary breast cancer surgery - Systematic screening as a tool for individualized rehabilitation: The ReScreen study
Countries of recruitment	Sweden
Health condition(s) or problem(s) studied	Breast cancer rehabilitation
Intervention(s)	Intervention group: Patients with high distress receive individualized rehabilitation including evaluation of individual needs and based on that physical, psychological or social interventions to promote rehabilitation. Control and observational groups: care as usual
Key inclusion and exclusion criteria	Inclusion criteria: Primary breast cancer, ≥18 years old, ability to communicate in Swedish. Exclusion criteria: Recurrent disease, pregnancy, cognitive impairment, severe mental illness and drug addiction.
Study type	Interventional. Allocation: Randomized, 3-armed. Masking: Blinded at allocation level. Primary purpose: Optimized rehabilitation
Date of first enrolment	May 2019
Target sample size	950
Recruitment status	Recruiting
Primary outcome(s)	Distress
Key secondary outcomes	Quality of Life, anxiety and depression, resilience, physical activity, health related behaviours, care satisfaction, health care utilization and sickness absence, patient and relatives experiences
Ethics Review	Regional Ethical Review Board in Lund, Sweden (reference number 2015/505)
Individual participant-level data (IPD) sharing statement	The IPD will not be shared

#### Eligibility criteria and randomization

**Fig. 3:**
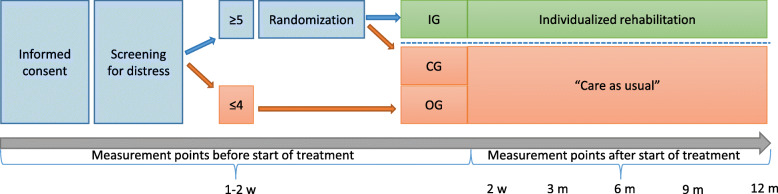
Overview of allocation and data collection points

Women diagnosed with primary BC with planned surgical or neoadjuvant treatment (as well as their relatives in the focus group and the health economical evaluations) who meet the inclusion criteria (Table 2) are recruited by a contact nurse. All patients are included 1–2 weeks after the cancer diagnosis. Inclusion is based on screening of distress using the Distress Thermometer [28], with a cut-off set at ≥5 on the 0–10-point scale. Patients identified as having increased distress (≥5) are randomized to the control (CG) or intervention group (IG) by a dedicated research nurse while patients identified as not having increased distress (≤4) form an observational group (OG) that will be followed longitudinally for comparison (Fig. 3). Prior to the study start a computerized random sequence was generated by an external statistician to ensure allocation concealment with a 1:1 ratio for the CG vs IG. Blinding is possible only at the allocation level.

**Table 2:** Inclusion and exclusion criteria of the ReScreen study

	Inclusion criteria	Exclusion criteria
Patients	Primary breast cancer	Recurrent disease
	≥18 years old	Pregnancy
	Ability to communicate in Swedish	Cognitive impairment
		Severe mental illness
		Drug addiction
Relatives	≥18 years old	Cognitive impairment
	Ability to communicate in Swedish	Severe mental illness

#### Sample size

Approximately 40% of the included patients are expected to suffer from increased distress (scoring ≥5 on the 0–10-point Distress Thermometer) and therefore to be available for randomization. A power calculation with distress as the primary outcome indicated that, with an expected reduction in distress of 1.0, a standard deviation (SD) of 2.9 and the significance level set at 0.05, approximately 300 patients are needed to achieve 80% power. When considering the proportion of patients scoring ≤4 on the Distress Thermometer and adjusting for expected dropout, a total of 950 patients will need to be included to ensure relevant power. Inclusion was started in May 2019 and will be running for approximately 5 years with an expected recruitment rate of 200 patients annually.

#### Control and observational groups

Patients included in the OG and patients randomized to the CG will receive care as usual, including follow-up by a surgeon/oncologist, contact with the contact nurse and, where appropriate, contact with a social worker or physiotherapist. Follow-up is mainly structured based on medical indicators/the treatment regime.

#### Intervention group (IG)

Patients randomized to the IG will, in addition to “care as usual”, be offered an individualized rehabilitation plan based on the decision support tool developed in phase I. The decision support tool is structured with focus on patients’ health-related behaviours (e.g. nutrition, tobacco and alcohol consumption, and exercise) and on clinically and evidence-based knowledge about known problem areas for patients with BC. These areas are practical/relational problems (e.g. family or work-related problems), physical problems (e.g. fatigue or pain) and psychological/emotional and existential problems (e.g. worry, anxiety, and spiritual or religious problems).

**Fig. 4:**
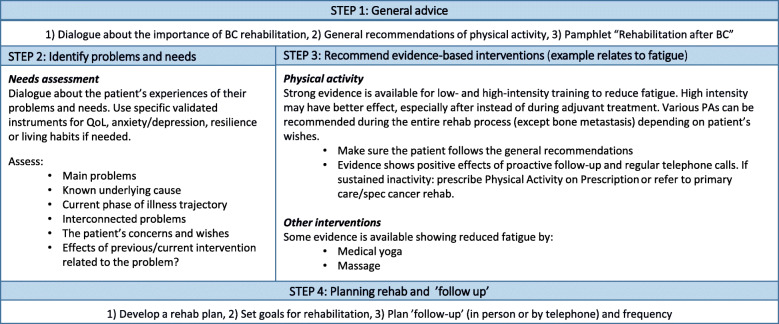
Example of the decision support tool focusing on problem area fatigue

The decision support tool will be used as a support in the dialogue between the patient and a dedicated research nurse and is structured in four steps. In the *first step* all patients will receive general advice about rehabilitation and exercise. The *second step* will focus on identifying patients’ individual needs, which in the *third step* will be matched to evidence-based interventions. In the *fourth step* the patient and the research nurse will decide on a rehabilitation plan with explicit goals for the rehabilitation process. They will also decide how the most effective follow-up can be organized. The rehabilitation plan will be evaluated based on the patients’ individual needs but as a minimum at 2 weeks after start of treatment and once a month during the first 3 months. The research nurse will act as a continuous health care contact and will be responsible for proactively and systematically evaluating the patient’s needs to ensure that potential new problems or changed rehabilitation needs are identified throughout the 1-year follow-up period and to promote retention and completeness of follow-up. An example of how the decision support tool works within the problem area of “fatigue” is given in Fig. 4.

#### Safety and ethical considerations

There is no potential risk of participating in this study as no participants will be deprived of any treatment or routine care. Any adverse events will be recorded. The project has been approved by the Regional Ethics Review Board in Lund, Sweden (reference number 2015/505 and 2018/924). All participants receive oral and written information about the study and are assured that participation in this study is voluntary and that they can withdraw from the study at any point without any effect on their before by a contact nurse treatment signing informed consent. All data will be stored securely and kept confidential. In case of substantive changes to the study protocol this will be communicated to relevant stakeholders. The trial results will be disseminated through publication in scientific journals regardless of the magnitude or direction of effect. Publications will follow the defined authorship criteria from the International Committee of Medical Journal Editors.

#### Data collection

Four data sources are used for data collection. Demographic and medical/treatment-related data are retrieved from the patients’ medical record. Self-reported data are collected using validated instruments at inclusion (1–2 weeks after cancer diagnosis, filled in at the outpatient centre) and 2 weeks and 3, 6, 9- and 12-months post start of treatment. Instruments are sent to patients by mail together with a pre-stamped envelope; and two reminders will be sent. Data collection are also conducted through national register data and through interviews with patients and relatives.

#### Demographic and medical/treatment-related data

Demographic, medical and treatment related data include information on age, living arrangements, marital and work status, level of education, body mass index, cancer stage, tumour characteristics, type of surgery and adjuvant treatment regimen including radiotherapy.

#### Self-reported data

*Distress* will be used as the primary outcome and is assessed using the Distress Thermometer [28]. The instrument consists of an 11-point numeric scale ranging from 0 to 10 (0 = no distress; 10 = extreme distress) and a problem list comprising 35 common problems in cancer patients, divided into five categories: practical problems, family-related problems, emotional problems, spiritual or religious problems, and physical problems, answered with “Yes” or “No”. The Distress Thermometer has been shown to be valid [11, 35] and have good potential as a screening tool for distress-related practical, family, emotional and physical problems in patients with cancer in a Swedish population [28, 35].

*Quality of life* will be evaluated using European Organization for Research and Treatment of Cancer (EORTC)-validated instruments including the general QoL instrument, QLQ-C30 v3.0 [36], and its BC-specific module, QLQ-BR23 [37]. The instruments consist of symptom and functional scales and are answered on 4 and 7-point Likert scales.

*Anxiety and depression* will be evaluated using the Hospital Anxiety and Depression Scale (HADS) [38] containing 14 questions answered on a 4-point Likert scale (0–3). The scale has shown sound psychometric properties in women with BC [39].

*Resilience* will be evaluated using the Connor-Davidson Resilience Scale (CD-RISC) [40] incorporating 25 items answered on a 5-point Likert scale (0–4). The scale has good psychometric properties [41] and is shown to be valid and reliable in a Swedish population [42].

*Physical activity and health-related behaviours* will be evaluated using single items collected from validated instruments from the Swedish National Board of Health and Welfare. Items related to physical activity will include questions about exercise and activity, while items related to health behaviours will include questions on tobacco and alcohol consumption, and nutrition.

*Care satisfaction* will be evaluated using single items inspired by a evaluation form related to care process, support and the comprehensiveness of the system developed by the Regional Cancer Centre (RCC), Sweden.

#### National register data

Health economic evaluations will be conducted based on register data and focus on health care utilization and sickness absence. Data regarding health care utilization within public inpatient and outpatient care will be collected from the National Patient Register, and data regarding sickness absence from the Swedish Social Insurance Agency.

#### Focus group interviews

Focus group interviews aiming to illuminate patients’ and their relatives’ experiences of the rehabilitation process after participation in the RCT study will be conducted to provide a deeper understanding of the effect of the RCT. Aiming for maximum variation related to age, type of treatment and group (IG or CG), a purposeful sample of patients and relatives will be invited to participate. Three focus group interviews per participant category (patients from the IG, and the CG, and their relatives) will be conducted using a semi-structured interview guide. The interviews will be audio-recorded and transcribed verbatim. Data will be analysed using conventional qualitative content analysis [34].

#### Data management and monitoring

An electronic case report form (eCRF) is used for registration of demographic and medical/treatment-related data. All data are to be completed in the eCRF within 2 months of the follow-up contacts. Self-reported instrument data are collected and stored in a system for instrument data. Data monitoring is performed inspired by the Good Clinical Practice (GCP) guidelines. In the pilot study (*n* = 90) and with the first 30 patients included in the full-scale study, completeness of data was monitored for all patients to ensure that potential systematic errors would be identified. Monitoring for all subsequent patients will be conducted twice a year. Data on items identified as specifically important, e.g. Distress Thermometer score, informed consent and randomization, will be monitored for all patients, while all other included data will be monitored for every 10th patient. Data monitoring will be performed by an independent monitor. No data monitoring committee (DMC) will included due to the characteristics of the study and local standards. The eCRF data, instrument data and register data will be merged when data collection is closed. Only the research group members will have access to the full trial dataset.

#### Analytical plan

Statistical analyses based on data level and data distribution will be conducted using the IBM SPSS Statistics for Windows, Version 21.0 (IBM Corp, Armonk, NY, USA). An intention-to-treat analysis will be adopted to manage missing data and imputation of missing values will be conducted according to the instructions for each instrument. For health economic evaluations, the number of full-time equivalents (whole days of sick leave/patient) will be calculated in the first year after cancer diagnosis. Furthermore, the mean time from diagnosis to return to full-time work will be analysed, as a complement to full-time equivalents to illustrate a possible change in the dynamics of rehabilitation. An evaluation of the Distress Thermometer as an indicator for extended rehabilitation needs in the Swedish context will be conducted with the aim to establish the optimal cut-off for distress. Data from the Distress Thermometer and HADS will be used. A receiver-operating characteristic (ROC) analysis of distress, with HADS as the golden standard, will be conducted evaluating sensitivity, specificity and positive/negative predictive value.

## Discussion

The initial phases of this complex intervention study have created a solid base for the RCT, which has been pilot- and feasibility tested. This project has the potential to explore effective approaches to early identification and intervention in patients treated for BC with risk of developing poor outcome and unmet needs, which may have a beneficial impact for the patients as well as on health care resources. Despite extended research in the field of BC rehabilitation there is still a gap of knowledge regarding rehabilitation from a comprehensive perspective. This means that extended knowledge about how to identify patients with extended needs, and how individualized rehabilitation interventions can be practised, is fundamental for further development of BC rehabilitation. The ReScreen study builds on available knowledge about the effectiveness of specific interventions and will contribute to greater knowledge about the effectiveness of screening-based, individualized rehabilitation from a patient, relative and health economic perspective.

To our knowledge, there are no standardized methods for screening and meeting each individual’s rehabilitation needs after primary BC diagnosis. However, it has been suggested that by using a holistic, long-term approach integrating multi-professional and multi-disciplinary teams, cost-effective, patient-centred rehabilitative care can be achieved [43]. The present study has the potential to investigate several factors that may affect BC patients’ access to rehabilitation and identify important factors in implementing individualized rehabilitation.

Two studies have previously evaluated the Distress Thermometer as a screening tool to identifying patients suffering from moderate to severe distress [18, 24]. According to Ploos et al. [18] early screening has the potential to identify patients at highest risk of being chronically distressed, indicating that high distress could be used as an indicator for extended rehabilitation needs. The present study will contribute to knowledge of how to identify women experiencing high distress after BC diagnosis as an indicator of extended rehabilitation needs. By exploring the association between distress and self-reported or medical related data, the factors that explain the cause of high distress can be identified. Minimizing the negative effects of these factors will result in optimized rehabilitation.

Health economic aspects such as health care utilization and sickness absence are important when evaluating the effect of intervention studies. However, these aspects are sparsely evaluated when testing the effect of rehabilitation interventions. This means that an important piece of knowledge is missing when laying the ground for health care development and implementation. To our knowledge, this study is the first that will provide health economic evaluations of the effect of early identification of rehabilitation needs and individualized rehabilitation focusing on health care utilization and sickness absence – information that is important for future development towards individualized rehabilitation within the BC area.

This study focuses on developing and evaluating a clinically relevant model that is feasible in today’s complex health care system. Through these efforts, we provide an intervention that, if shown to be effective, can be implemented in clinical practice. This means that this project could benefit patients at an early stage after study evaluation and may in the near future change clinical practice.

## Data Availability

Not applicable.

## Abbreviations

BC: Breast cancer
CD-RISC: Connor-Davidson resilience scale
CG: Control group
eCRF: Electronic case report form
EORTC: European Organisation for Research and Treatment of Cancer
GCP: Good clinical practice
HADS: Hospital anxiety and depression scale
HCP: Health care professional
IG: Intervention group
OG: Observational group
QoL: Quality of life
RCC: Regional Cancer Centre
RCT: Randomized controlled trial
ROC: Receiver-operating characteristic
SD: Standard deviation
